# Retrospective parental assessment of childhood neurodevelopmental problems: the use of the Five to Fifteen questionnaire in adults

**DOI:** 10.1192/bjo.2019.30

**Published:** 2019-05-17

**Authors:** Tove Lugnegård, Susanne Bejerot

**Affiliations:** Doctor, Centre for Psychiatry Research, Department of Clinical Neuroscience, Karolinska Institutet, Sweden; Doctor, School of Medical Sciences and the University Health Care Research Centre, Faculty of Medicine and Health, Örebro University, Sweden

**Keywords:** Autistic spectrum disorders, attention deficit hyperactivity disorders, tic disorders, rating scales, developmental disorders

## Abstract

**Background:**

Attention-deficit hyperactivity disorder and autism are increasingly recognised in adults. For a diagnostic evaluation, parental information on childhood development is needed. However, no instruments that retrospectively describe neurodevelopmental problems in childhood are validated for evaluating adults. The 181-item parent-report questionnaire Five to Fifteen (FTF) is nevertheless frequently used for assessments in adulthood.

**Aims:**

To examine if FTF is reliable for obtaining retrospective neurodevelopmental history among young adults.

**Method:**

Details of parents who had assessed their children with the FTF for neuropsychiatric evaluation were retrieved and they were asked to complete the FTF again 10–19 years later. Agreements between original and retrospective scorings were analysed.

**Results:**

Long-term reliability for FTF varies considerably between individual items. Several difficulties are reported as more severe at the retrospective scoring than at the original scoring. A selection of 24 items (FTF-Brief) with good agreement over time, is presented for use in adult psychiatry settings.

**Conclusion:**

Neuropsychiatric symptoms may fluctuate over time and become more prominent when demands increase. Informants' recollections of their child's neurodevelopmental symptoms may be a selection of symptoms that are longstanding rather than present at a specific age in childhood.

**Declaration of interest:**

None.

Attention-deficit hyperactivity Disorder (ADHD) and autism spectrum disorder (ASD) are neurodevelopmental conditions typically identified in childhood. However, these diagnoses are increasingly recognised in adult psychiatric settings.^1–3^ A considerable proportion of individuals are not diagnosed in childhood, but seek psychiatric services as adults,^4,5^ which has led to a markedly increased demand for neuropsychiatric evaluations in adulthood. Assessments in adults pose some problems compared with childhood assessments. Diagnostic criteria for ADHD and ASD imply that difficulties must have been present at an early age.^6,7^ Consequently, it is vital to obtain detailed information on childhood development, when diagnosing neurodevelopmental disorders. Adults with ADHD and ASD have been shown to be poor reporters of their internal states such as inattention or impulsivity.^8,9^ Subsequently, additional information is needed for a diagnostic evaluation.

The Five to Fifteen (FTF) parent questionnaire is an instrument for obtaining a developmental history of a child aged between 5 and 15 years.^10^ It was developed by Nordic experts in child psychiatry during the 1990s with the specific purpose of providing a picture of a child's unique pattern of strengths and difficulties. Although the FTF was developed solely for paediatric assessments, it is frequently used in adult psychiatric settings to collect retrospective information from parents on the developmental difficulties of the offspring. Since there are no validated and well-studied instruments for this purpose, such ‘off-label’ use of the FTF has been widely accepted in the Nordic countries. The aim of the study is to examine to what extent the parent questionnaire FTF is a reliable instrument for obtaining retrospective neurodevelopmental history among young adults. Our second aim is to provide guidance for future use of the FTF in adult clinical settings.

## Method

### Measurement

The FTF is a parent-report questionnaire consisting of 181 items describing neurodevelopmental problems that affect daily functioning. There are no algorithms for specific diagnoses according to DSM or ICD, but rather a comprehensive approach of screening for signs and symptoms in a broad range of domains. The clinical validity in a child population is shown to be good.^10–12^

The items are organised in eight general domains: motor skills, executive functioning, perception, memory, language, learning, social skills and emotional/behavioural problems. The eight general domains are then subdivided into 22 subdomains. Each of the 181 statements can be endorsed as: does not apply, 0; applies sometimes or to some extent, 1; or definitely applies, 2.

The present FTF is the result of several draft versions used from 1995 to 2003. Adaptations were made in the first years of its use, which has resulted in an addition of in total 37 items and re-arrangement of existing items. From 2004 onwards, the present version has been used. However, the basic structure as well as the vast majority of items are identical for all versions. For details, see Kadesjö^10^ and Trillingsgaard.^12^

### Procedure and participants

Case reports from individuals born between 1984 and 1993, assessed by a multidisciplinary team at the Neuropsychiatric Clinic for Children and Adolescents (NCCA) in Karlstad, Sweden, between 1995 and 2005, were examined. The NCCA was, during this period, an out-patient clinic specialised in evaluation of ADHD, ASD and other developmental conditions in children and adolescents under the age of 19. Diagnoses were made by consultant psychiatrists with expertise in neurodevelopmental disorders and according to DSM-IV.

**Fig. 1:**
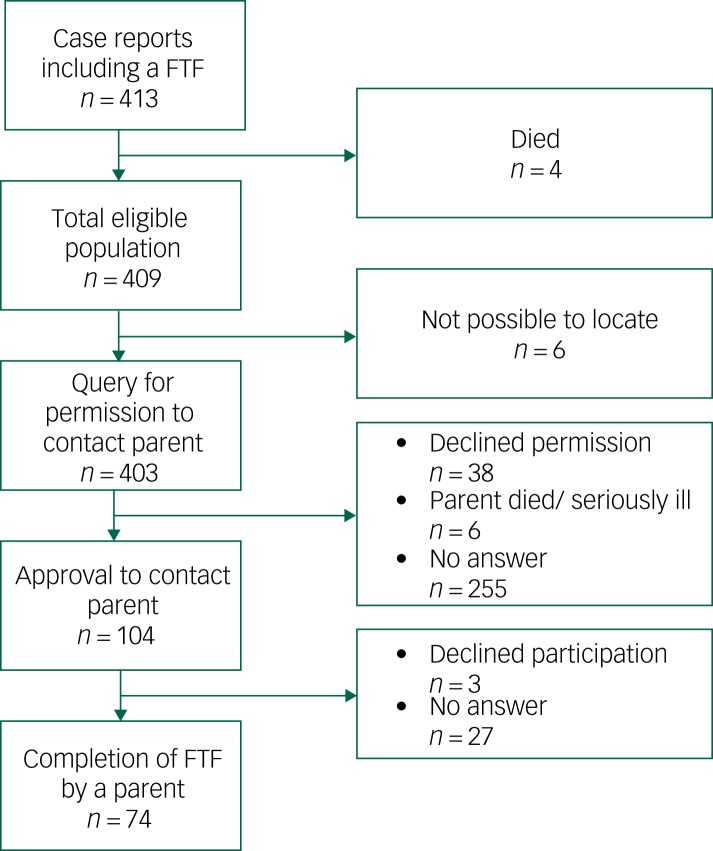
Flow chart for participation.

The NCCA was a regional centre within the public health services, free of charge and with a catchment area that included the whole county of Värmland (population 280 000). Children were referred from school healthcare, primary care or by self-referrals through parents. Possible study participants were individuals (a) older than 5 years and younger than 13 years at time of assessment and (b) with a complete FTF present in the case report. The age range was chosen in order to minimise assessments completed during puberty and teens. A query was sent to ask for permission from the individual to contact the parent(s) for a second assessment using the FTF. After written consent was obtained, the FTF questionnaire was sent to the same parent who completed the original FTF along with an information letter. Parents were asked to answer the questionnaire in a retrospective way, i.e. to describe their offspring as he/she was as a child at the age of the original assessment. In cases of no reply, a reminder was sent 3–4 weeks later. This stepwise procedure resulted in 74 pairs of FTFs (one original, completed in childhood, and one retrospective, completed in adulthood for the same individual) (Fig. 1).

FTF, Five to Fifteen.

Characteristics of the 74 study participants are presented in Table 1. There were no significant differences concerning age and gender between the study sample and the total eligible population. A total of 58 (78%) of participants had obtained at least one neurodevelopmental diagnosis after the assessment had been completed. There were 27 individuals (36%) who received a diagnosis of ADHD as their principal diagnosis and 24 individuals (32%) with ASD as their principal diagnosis. Two were primarily diagnosed with mild intellectual disability, two with Tourette disorder and three with dyslexia. One individual was diagnosed with obsessive–compulsive disorder only, and, in six individuals (8%), marked developmental difficulties were identified without, however, fulfilling criteria for a specific psychiatric diagnosis. Nine individuals (12%) did not receive any psychiatric diagnosis after assessment.

**Table 1:** Characteristics of study sample (*n* = 74)

Characteristic	Value
Gender, *n* (%)	
Male	58 (78)
Female	16 (22)
Age at original scoring, years: mean (s.d.) range	8.5 (2.3) 5–12
Age at second scoring, years: mean (s.d.) range	24.5 (2.5) 21–30
Time between scorings, years: mean (s.d.) range	15.9 (1.7) 10–19
Informant(s), *n* (%)	
Mother	29 (39)
Father	3 (4)
Both parents	42 (57)

### Analysis

Analysis of agreement was performed by applying the scorings in two different modes: (a) by collapsing score 1 and 2 into the same category (any difficulties), accordingly obtaining a dichotomous scale: no difficulties, 0 or any difficulties, 1–2 (0/1–2), and (b) by using the full Likert scale: no difficulties, 0; some difficulties, 1; marked difficulties, 2 (0/1/2). Agreement between original scoring (time 1) and retrospective scoring (time 2) was analysed by calculating for each item: (a) percentage of equal scores both times and (b) kappa value (dichotomous scale) or weighted kappa value (full Likert scale).^13^ Two items (item 110 and 111) were excluded from analysis as they do not imply a scoring of behaviour, but only request descriptions of knowledge and skills.

### Ethics

The study was approved by the Medical Ethical Review Board in Uppsala, Sweden, 2014/172.

## Results

Supplementary Table 1 details the results of FTF on an item-by-item basis (available at https://doi.org/10.1192/bjo.2019.30). A total of 26 participants were originally assessed with early (shorter) versions of the FTF and, consequently, items absent in these versions were not available for comparisons, which results in *n* lower than 74 for these items as shown in the footnote supplementary Table 1. Distribution of FTF scores.

Distribution of original and retrospective scoring for each item is shown in supplementary Table 1. Any difficulties (scores of 1 or 2) at the original assessment were reported for: gross motor skills 50%; fine motor skills 40%; attention 70%; hyperactive/impulsive 50%; hypoactive 50%; planning/organising 70%; relation in space 30%; time concepts 50%; body perception 30%; visual perception 15%. Memory 30%; language comprehension 40%; expressive language skills 30%; communication 40%; reading/writing 60%; maths 40%; general learning 40%; coping in learning 60%; social skills 30%; internalised emotional/behavioural problems 25%; externalised emotional/behavioural problems 40%; obsessive–compulsive problems 20%.

### Agreement between original and retrospective scorings

Agreement between original and retrospective scoring for each item is shown in supplementary Table 1. ‘Equal score both times’ indicates the percentage of participants with the same score both times. Agreement is presented both dichotomously (0/1–2) and for the full use of the scale (0/1/2). An overview of kappa value interpretation is shown in Table 2.^13^

**Table 2:** Interpretation of Cohen's kappa

Value of kappa	Level of agreement
<0.20	Poor
0.21–0.40	Weak
0.41–0.60	Moderate
0.61–0.80	Substantial
0.81–0.1	Almost perfect

Agreement varies between subdomains as well as between individual items. For most items, dichotomous use of scale renders a higher kappa value compared with the full use of the scale. For the subdomain gross motor skills, agreement is moderate to substantial except for one item (item 4). For the subdomain fine motor skills, agreement is weak to moderate.

Agreement is moderate on the majority of items for the subdomain attention; however, four items show weak agreement (items 19, 21, 25, 26). For the subdomain hyperactive/impulsive, agreement is moderate to substantial with the exception of one item (item 35), which shows weak agreement. For the subdomain hypoactive, agreement is moderate. Two items of the three-item subdomain planning/organising show moderate agreement, whereas one item (item 40) shows weak agreement. For the subdomains relation in space and time concepts, agreement is generally weak. For body perception, two items show substantial agreement (items 52 and 54), however, other items present weak to moderate agreement. For the subdomain visual perception, agreement is weak to moderate.

For the subdomain memory, agreement is weak to moderate. Moderate agreement is seen for the subdomain language comprehension. For the subdomain expressive language skills, agreement is weak to moderate. The three items forming the subdomain communication show weak to moderate agreement. For the subdomains reading/writing and maths, agreement is moderate to substantial. General learning and coping in learning show moderate to substantial agreement, with the exception of three items (items 108, 117 and 121), which show weak agreement. The subdomains social skills and internalised emotional/behavioural problems show weak agreement. For the subdomains externalised emotional/behavioural problems and obsessive–compulsive problems, agreement is weak to moderate.

For two items (items 57 and 86) the kappa value is around 0 even though the percentage with an equal score on both occasions is high (86% and 98%, respectively), which is a consequence of a much-skewed distribution of scorings for these items.

### Abridge version of the FTF

In total, 24 informative items with acceptable psychometric properties, representing 18 of the 22 subdomains in the FTF, were selected for an abridged version of the FTF. The selected items are shown in the Appendix. The abridged FTF (FTF-Brief) is available in English and Swedish in supplementary Appendices 1 and 2, respectively.

## Discussion

To our knowledge, this is the first study in which an instrument that assesses a wide range of neurodevelopmental and behaviour problems in childhood is examined for retrospective use in adulthood. By letting parents retrospectively reassess the offspring with the same questionnaire – the parent questionnaire FTF – more than a decade after the original assessment we could show that a subset of the FTF items are indeed reliable measures of childhood symptoms.

In this study, 74 children were assessed for neuropsychiatric disorder, which included the parent questionnaire the FTF. The highest prevalence of difficulties was seen in the FTF subdomains attention and planning/organising, followed by the subdomains gross motor skills, hyperactive/impulsive, hypoactive, time concepts, reading/writing and coping in learning, which presumably reflects the most common reasons for referral to a neuropsychiatric evaluation in childhood. The great majority of the children were diagnosed with ADHD and/or ASD.

### Retrospective scoring

When the child had reached young adulthood (i.e. 10–19 years after the original scoring) the same parent scored FTF a second time, according to how the parent recalled their child's behaviours at the age of the original assessment. In order to test time reliability for FTF, we measured agreement between scorings in child- and adulthood. A considerable variation between individual items was found across all subdomains, however, it was more pronounced in some. For the majority of subdomains, higher scores (i.e. more severe difficulties) are reported at the retrospective assessment. Possibly, parents are likely to report marked symptoms that have developed later on, regardless of presence at the original assessment. Neuropsychiatric symptoms may fluctuate over time and become more prominent when demands increase, which presumably is the case when coming of age. In contrast, for the subdomains hyperactive/impulsive and externalised emotional/behavioural problems, scores were generally lower at the retrospective assessment, which may be explained by the well-known reduction with age of these problems in ADHD.^14^

What could explain the variation on agreement? First, items differ entirely in characteristics: some being concrete and more easily recalled (for example ‘hoarse voice’, ‘stutters’), others being vague and requiring thoughtful judging (for example ‘difficulty understanding or using abstract terms’). Second, parents were asked to recall symptoms the child had at a certain age, i.e. the exact age at the original assessment. However, several items reflect symptoms that can be assumed to vary considerably over time (such as ‘does not like reading’, ‘has poor appetite’), and, therefore, difficult to score retrospectively for a specific age. Moreover, new aberrant behaviour may emerge over time, whereas others will aggravate or diminish. Clearly, it may be challenging for parents to recall, after a decade or two, how their child appeared at an exact age. However, when using the FTF in an adult clinical setting, the exact age of the presence of a specific behaviour is usually of little interest: the aim is rather to find out the trajectory of difficulties and what difficulties were ever present during childhood. Applied in this way, the FTF used in retrospect is presumably more valid than implied by the kappa scores in our study.

### Informants’ reports or self-reports in the diagnostic work-up

Previous research regarding the accuracy of retrospective recall in neurodevelopmental disorders has been somewhat equivocal. Some researchers claim that the adult patient with ADHD appears to be the best informant about symptoms of ADHD.^14^ In contrast, other studies in adults show that self-reports of ADHD and ASD are less reliable than informant reports.^8,9,15–17^ To recall one's own behaviour in childhood is a difficult task for any adult, but even more so for adults with neurodevelopmental disorders; ADHD and ASD are both associated with memory impairments.^18,19^ However, individuals with more severe current symptoms are shown to be more likely to report having had symptoms during childhood. There is evidence to suggest that severity of childhood symptoms predicts more accurate recall.^20^

The gold standard for diagnostic assessments of ASD includes the Autism Diagnostic Interview – Revised (ADI-R) or Diagnostic Interview for Social and Communication Disorders (DISCO). These are comprehensive parental interviews, which take approximately 3–4 h to complete. ADI-R and DISCO are similarly to FTF aimed at use in paediatric settings; however, they are frequently administrated in adult psychiatric settings. We are not aware that they have been validated for retrospective use in parental interviews of adults. There is an absence of a defined gold standard for diagnosing ADHD, but behavioural rating scales, clinical observations and informant reports are recommended.

### FTF-Brief

The large number of items (181) limits the use of the FTF when administered in retrospect: both because of the variation on agreement shown in our study and because some parents are unwilling or unable to fill in extensive questionnaires, and others are solely available for a short phone interview. For these reasons, a selection of items was combined into an abbreviated version of the FTF, the FTF-Brief (supplementary Appendices 1 and 2).

The 24 items in the FTF-Brief are representative for neurodevelopmental problems as such, cover a wide range of symptoms and importantly, frequently yield affirmative responses. Thirteen out of the 24 items were confirmed by a majority of the parents and only 2 items (item 60 ‘difficulty managing jig-saw puzzles’; item 174 ‘compulsively repeating certain activities’) were confirmed by less than a third of the parents at the original assessment. Notably, since the selected items show moderate to substantial agreement between the two assessment points, the parents are likely to remember them. In a follow-up study of ADHD symptoms in adults diagnosed with ADHD in childhood, six symptoms demonstrated high discriminating power in differentiating the individuals with ADHD from healthy controls.^21^ Four of these items are also included in the FTF-Brief (item 18 ‘fails to give close attention to details’; item 25 ‘is easily distracted’; item 27 ‘fidgets with hands or feet’; item 31 ‘is often on the go’). Several ASD symptoms that are reported to persist into young adulthood (such as inappropriate emotional response to age peers; inappropriate quality of interaction; maintenance of sameness in routines)^22^ correspond nicely to three items in the FTF-Brief (item 131 ‘is perceived by age peers as odd’; item 135 ‘says socially inappropriate things’; item 147 ‘is very upset by tiny routine change’).

Four of the FTF subdomains were not included in the FTF-Brief (i.e. relation in space; time concepts; general learning; internalised emotional/behavioural problems) because of few affirmative responses and/or low agreement between the two assessments. Internalised emotional problems are known to improve with age in ASD^23^ and thus may be forgotten. In ADHD, internalising problems are mainly reported as a comorbidity in girls and therefore of less importance when assessing boys,^24^ which may explain the low kappa value for these items. In sum, we believe that the FTF-Brief is suitable for use in adult clinical settings and for research.

### Limitations

Our study has some important limitations. Only 25% of eligible participants accepted our request to send the FTF to a parent and the majority (63%) did not answer the request at all. Persisting executive dysfunction because of a neurodevelopmental diagnosis may well have contributed to the low response rate. Conflicts between the parent and the adult offspring could be another reason; the request to participate in the study may never have reached the parent. However, among parents who were contacted, 71% were willing to fill out the FTF, which is a high response rate. Moreover, in the majority of cases both parents collaborated in filling out the FTF. The majority of our participants were diagnosed with ADHD and/or ASD in childhood. Consequently, it remains unknown whether the FTF is valid for assessing childhood symptoms retrospectively in individuals diagnosed with other psychiatric conditions in childhood. However, the presumably non-neurodevelopmental psychiatric disorders, i.e. other than ASD, ADHD and chronic tic disorder, do not require a childhood onset according to current diagnostic criteria.

### Implications

The FTF was developed to assist in the assessment of children with a suspected neurodevelopmental disorder. It is also currently used to collect information from parents on childhood behaviour in retrospect, when an adult is examined for ADHD or ASD. Our study shows considerable variation for the 181 FTF items on agreement between parental original and retrospective scoring, thus implying some items are more reliable over time than others. When an adult is investigated for behavioural problems in childhood, in order to determine if diagnostic criteria for ADHD or ASD can be fulfilled, the exact age for the presence of a specific problem is of minor interest. Rather it is the identification of neurodevelopmental symptoms in childhood present before a certain age, which is of importance. In this endeavour, the FTF seems to be helpful. In total, 24 items showed good agreement between both assessments, in addition to providing good coverage for a wide range of symptoms commonly observed in neurodevelopmental disorders. These 24 items are suggested as useful in adult psychiatry settings, and thus selected to constitute the short version of the FTF, the FTF-Brief. Irrespective of applying the FTF-Brief or the full FTF, it is not advisable to replace a clinical parental interview solely with a questionnaire.

According to our clinical experience in adult clinical settings, the full FTF is a valuable tool. It prepares the parent and the adult child for the retrospective interview and encourages a mutual understanding for the life-long problems that define ADHD and ASD. However, because of the considerable variation in agreement for the 181 FTF items between original and retrospective scoring, the full FTF cannot be recommended for retrospective use in clinical research.

## Supplementary material

For supplementary material accompanying this paper visit http://dx.doi.org/10.1192/bjo.2019.30. click here to view supplementary material

## Appendix

**Appendix:** Five to Fifteen (FTF)-Brief: 24 items representing 18 of the original 22 subdomains

Item number	Original item number	Item definition
		*Gross motor skills*
1	1	Difficulty acquiring new motor skills
2	3	Difficulty running fast and smoothly
		*Fine motor skills*
3	12	Difficulty using knife and fork
		*Attention*
4	18	Fails to give close attention to details
5	25	Is easily distracted
		*Hyperactive/impulsive*
6	27	Fidgets with hands or feet
7	31	Is often ‘on the go’
		*Hypoactive*
8	37	Difficulty completing tasks
		*Planning/organising*
9	41	Difficulty planning completion of tasks
		*Body perception*
10	52	Does not care about the fit of clothes
		*Visual perception*
11	60	Difficulty managing jig-saw puzzles
		*Memory*
12	69	Difficulty learning things by rote
		*Language comprehension*
13	72	Difficulty understanding explanations/instructions
		*Expressive language skills*
14	85	Difficulty pronouncing complex words
		*Communication*
15	90	Difficulty explaining what has happened
		*Reading/writing*
16	94	Difficulty understanding what he/she is reading
		*Math*
17	102	Difficulty with math problems formulated as written text
		*Coping in learning*
18	116	Difficulty completing tasks
		*Social skills*
19	131	Is perceived by age peers as odd
20	135	Says socially inappropriate things
21	147	Is very upset by tiny routine change
		*Externalised emotional/behavioural problems*
22	161	Loses temper
23	164	Teases others
		*Obsessive–compulsive emotional/behavioural problems*
24	174	Compulsively repeating certain activities
