# Supplementary material for: Retrospective parental assessment of childhood neurodevelopmental problems: the use of the Five to Fifteen questionnaire in adults
Source: BJPsych Open. 2019 May 17;5(3):e42. doi: 10.1192/bjo.2019.30 (PMC6537455; doi:10.1192/bjo.2019.30)
Supplement: Supplementary file 1 [file bjosup.zip › S2056472419000309sup002.pdf]

Supplementary Table 1. Distribution of scores at time point 1 and time point 2 and agreement between scorings

em #	Item definition	Distribution		Agreement			
		Any difficulties (score 1 or 2)		Dichotomous use of scale (0/1–2)		Full use of scale (0/1/2)	
		Original scoring %	Retrospective Scoring %	Equal score both times %	Kappa	Equal score both times %	Weighted kappa
	Gross motor skills						
	Difficulty acquiring new motor skills	53	54	82	0.64	61	0.47
	Difficulty throwing and catching a ball	49	60	78	0.56	54	0.40
	Difficulty running fast and smoothly	56	65	85	0.68	64	0.53
	Difficulty/does not like to participate in game sports	58	76	71	0.36	49	0.35
	Has balance problems	42	52	75	0.51	54	0.34
	Often trips and falls	38	44	79	0.57	69	0.59
	Has clumsy movements	47	60	70	0.41	59	0.43
	Fine motor skills						
	Has difficulty drawing	37	44	70	0.38	64	0.49
	Difficulty manipulating small objects	26	46	66	0.28	57	0.22
	Difficulty pouring water into a glass	29	40	69	0.31	64	0.32
	Often spills food onto clothes when eating	46	51	78	0.56	60	0.42
	Difficulty using knife and fork	49	44	72	0.44	62	0.47
	Difficulty buttoning buttons/tying shoe-laces	53	57	69	0.38	54	0.42
	Difficulty using a pen	38	49	77	0.53	68	0.55
	Has not developed clear hand dominance	15	18	85	0.46	81	0.50
	Writing is slow and awkward	53	60	66	0.32	50	0.34
	Has immature pen-grip	29	40	69	0.31	63	0.34
	Attention						

em #	Item definition	Distribution		Agreement			
		Any difficulties (score 1 or 2)		Dichotomous use of scale (0/1–2)		Full use of scale (0/1/2)	
		Original scoring %	Retrospective Scoring %	Equal score both times %	Kappa	Equal score both times %	Weighted kappa
[a]	Fails to give close attention to details	70	65	77	0.47	60	0.50
[a]	Difficulty sustaining attention	83	75	79	0.38	56	0.36
	Does not seem to listen when spoken to	77	73	80	0.46	53	0.37
	Difficulty following through on instructions	73	79	77	0.38	48	0.30
[a]	Difficulty organising tasks	78	82	82	0.45	47	0.32
	Dislikes tasks requiring mental effort	77	80	80	0.41	52	0.36
[a]	Loses things	55	61	80	0.58	55	0.39
	Is easily distracted	74	75	74	0.31	58	0.39
[a]	Is forgetful in daily activities	69	69	73	0.38	42	0.24
	Hyperactive/impulsive						
	Fidgets with hands or feet	67	55	77	0.52	59	0.53
	Often leaves seat	72	57	74	0.45	58	0.50
[a]	Runs about or climbs excessively	49	43	81	0.62	72	0.63
[a]	Difficulty playing quietly	45	40	79	0.57	70	0.54
	Is often 'on the go'	55	44	84	0.68	68	0.61
[a]	Talks excessively	41	33	74	0.44	70	0.47
[a]	Blurts out answers	31	27	78	0.46	76	0.41
	Difficulty awaiting turn	63	46	80	0.61	66	0.50
[a]	Interrupts or intrudes on others	60	38	60	0.24	49	0.23
	Hypoactive						
	Difficulty getting started	67	78	78	0.46	49	0.31

em #	Item definition	Distribution		Agreement			
		Any difficulties (score 1 or 2)		Dichotomous use of scale (0/1–2)		Full use of scale (0/1/2)	
		Original scoring %	Retrospective Scoring %	Equal score both times %	Kappa	Equal score both times %	Weighted kappa
	Difficulty completing tasks	60	71	77	0.50	51	0.35
	Daydreams	50	53	75	0.50	61	0.52
	Is slow or lacking energy	32	39	76	0.49	67	0.42
	Planning/organising						
	Difficulty understanding consequences	62	65	66	0.27	51	0.37
	Difficulty planning completion of tasks	65	78	80	0.52	55	0.40
	Difficulty completing complex task	76	79	82	0.49	59	0.40
	Relation in space						
	Difficulty finding the way around	19	30	74	0.31	68	0.33
	Feels disconcerted by height discrepancies	17	24	80	0.40	77	0.46
	Difficulty judging distance or size	34	40	73	0.43	63	0.40
	Difficulty knowing how to rotate things	45	39	67	0.33	54	0.29
	Bumps into other people	31	34	77	0.48	66	0.36
	Time concepts						
	Has poor time concepts	62	59	65	0.26	53	0.34
	Has vague ideas of what time it is	36	50	62	0.24	53	0.24
	Repeats questions about time	57	57	58	0.15	48	0.20
	Difficulty understanding time concepts	34	53	51	0.03	42	0.12
	Body perception						
	Does not care about the fit of clothes	44	46	85	0.69	68	0.55
	Poor perception of cold	25	53	67	0.35	57	0.33

em #	Item definition	Distribution		Agreement			
		Any difficulties (score 1 or 2)		Dichotomous use of scale (0/1–2)		Full use of scale (0/1/2)	
		Original scoring %	Retrospective Scoring %	Equal score both times %	Kappa	Equal score both times %	Weighted kappa
	Poor body awareness	31	44	84	0.66	75	0.60
	Is oversensitive to touch	38	49	65	0.30	54	0.32
	Difficulty imitating other people's movements	35	52	71	0.42	55	0.39
	Visual perception						
	Misinterprets pictures	3	12	86	−0.05	86	−0.04
	Difficulty perceiving difference of similar shapes	13	24	83	0.43	78	0.33
	Difficulty drawing concrete pictures	22	29	75	0.35	71	0.34
	Difficulty managing jig-saw puzzles	25	35	81	0.55	74	0.46
	Memory						
	Difficulty remembering personal data	28	29	79	0.49	74	0.44
	Difficulty remembering people's names	20	24	79	0.38	76	0.52
	Difficulty remembering names of days, months	37	28	76	0.46	73	0.47
	Difficulty remembering non-personal facts	31	50	65	0.29	54	0.32
	Difficulty remembering recently experienced events	49	49	71	0.43	63	0.47
	Difficulty remembering past events	21	35	71	0.29	65	0.21
	Difficulty remembering where he/she puts things	65	68	74	0.41	53	0.30
	Difficulty remembering times for meetings etc.	45	54	55	0.11	46	0.16
	Difficulty learning things by rote	40	59	76	0.53	61	0.50
	Difficulty remembering complex instructions	71	83	79	0.41	61	0.42
	Difficulty acquiring new skills	32	50	64	0.27	55	0.29
	Language comprehension						

em #	Item definition	Distribution		Agreement			
		Any difficulties (score 1 or 2)		Dichotomous use of scale (0/1–2)		Full use of scale (0/1/2)	
		Original scoring %	Retrospective Scoring %	Equal score both times %	Kappa	Equal score both times %	Weighted kappa
	Difficulty understanding explanations/instructions	47	64	77	0.55	59	0.46
	Difficulty following a story read out loud	23	40	77	0.48	69	0.40
	Difficulty understanding the meaning of what is said	46	51	71	0.41	62	0.42
	Difficulty with abstract concepts	48	60	70	0.41	55	0.39
	Tends to misinterpret what is said	30	48	72	0.43	64	0.42
	Expressive language skills						
	Is uncertain of speech sounds	35	38	77	0.50	70	0.44
	Difficulty learning the names of colours, people etc	19	26	76	0.34	74	0.33
	Difficulty finding the right words	35	20	74	0.36	68	0.31
	Tends to remember words incorrectly	19	24	84	0.52	84	0.57
	Difficulty explaining what he/she wants	38	38	78	0.53	69	0.51
	Difficulty speaking fluently	31	28	83	0.59	70	0.47
	Difficulty expressing in whole sentences	35	32	82	0.59	71	0.51
	Has specific speech problem	21	21	80	0.41	74	0.39
	Difficulty pronouncing complex words	44	40	79	0.56	69	0.50
[a]	Has a hoarse voice	2	0	98	0.00	98	0.00
	Stutters	3	4	96	0.38	95	0.27
	Speaks to fast	32	20	66	0.15	61	0.10
	Has muddled speech	28	31	72	0.33	65	0.29
	Communication						
	Difficulty explaining what has happened	43	47	75	0.50	62	0.48

em #	Item definition	Distribution		Agreement			
		Any difficulties (score 1 or 2)		Dichotomous use of scale (0/1–2)		Full use of scale (0/1/2)	
		Original scoring %	Retrospective Scoring %	Equal score both times %	Kappa	Equal score both times %	Weighted kappa
	Difficulty keeping 'on track' when telling	39	49	72	0.45	57	0.36
	Difficulty carrying on a conversation	39	52	64	0.28	54	0.28
	Reading/writing						
[b]	Difficulty acquiring reading skills	43	48	80	0.59	72	0.61
[b]	Difficulty understanding what he/she is reading	51	56	84	0.67	71	0.61
[b]	Difficulty reading a text loud	62	62	81	0.60	64	0.53
[b]	Does not like reading	51	65	78	0.56	71	0.58
[b]	Guesses when reading	52	58	79	0.57	65	0.56
[b]	Difficulty with spelling	60	63	79	0.56	58	0.51
[b]	Difficulty with handwriting	58	68	82	0.63	61	0.55
0[b]	Difficulty formulating in writing	57	62	75	0.49	58	0.50
	Math						
1[b]	Difficulty acquiring basic math skills	30	52	75	0.51	59	0.32
2[c]	Difficulty with math problems formulated as written text	62	69	80	0.56	62	0.55
3[c]	Difficulty applying various rules for counting	41	52	72	0.44	59	0.46
4[c]	Difficulty learning the tables of multiplication	49	56	88	0.77	70	0.58
5[b]	Difficulty performing mental counting operations	41	56	70	0.42	57	0.40
	General learning						
6	Difficulty understanding instructions	59	62	75	0.47	52	0.35
7	Difficulty understanding or using abstract terms	44	56	73	0.47	59	0.44

m #	Item definition	Distribution		Agreement			
		Any difficulties (score 1 or 2)		Dichotomous use of scale (0/1–2)		Full use of scale (0/1/2)	
		Original scoring %	Retrospective Scoring %	Equal score both times %	Kappa	Equal score both times %	Weighted kappa
8	Difficulty participating in discussions with age peers	47	63	60	0.22	43	0.17
9	Difficulty learning facts about environment	27	40	78	0.51	65	0.39
	Coping in learning						
2[d]	Difficulty planning and organising	79	84	84	0.48	50	0.31
3[d]	Difficulty shifting plan or strategy	80	90	86	0.49	56	0.34
4[e]	Difficulty applying adult's explanation	62	75	77	0.48	56	0.40
5[e]	Difficulty solving abstract tasks	54	68	82	0.63	68	0.60
6[e]	Difficulty completing tasks	62	58	83	0.65	62	0.51
7[e]	Is unmotivated for school or work	54	67	62	0.22	48	0.29
8[e]	Is slow in learning situations	56	63	71	0.40	56	0.42
9[e]	Does things too hastily	47	39	76	0.51	58	0.43
0[e]	Can not take responsibility for own actions	61	66	82	0.61	61	0.48
1[e]	Is very much in need of support	66	52	64	0.27	52	0.37
	Social skills						
2	Does not understand other people's social cues	38	71	55	0.18	41	0.20
3	Difficulty understanding other people's feelings	55	70	67	0.31	47	0.27
4	Difficulty taking account of other people's needs	58	55	67	0.33	52	0.30
5	Difficulty expressing emotions in words	33	66	49	0.08	36	0.04
6	Has monotone or 'different' voice	3	21	79	0.08	79	0.05
7	Difficulty expressing emotions in body language	14	52	51	0.04	48	0.03
8	Has an old-fashioned style	20	39	81	0.57	80	0.55

em #	Item definition	Distribution		Agreement			
		Any difficulties (score 1 or 2)		Dichotomous use of scale (0/1–2)		Full use of scale (0/1/2)	
		Original scoring %	Retrospective Scoring %	Equal score both times %	Kappa	Equal score both times %	Weighted kappa
9	Difficulty behaving in a way expected by age peers	64	73	72	0.35	47	0.26
0	Difficulty knowing how to behave socially	44	62	62	0.26	51	0.26
1	Is perceived by age peers as odd	56	70	77	0.51	59	0.43
2	Makes a fool of him/herself	21	33	70	0.25	64	0.19
3[f]	Seems to lack common sense	27	52	60	0.21	52	0.20
4	Has a weak sense of humour	14	29	71	0.17	67	0.13
5	Says socially inappropriate things	32	33	80	0.55	76	0.54
6	Difficulty following rules	58	57	69	0.36	57	0.32
7	Quarrels with age peers	52	48	70	0.41	59	0.37
8	Difficulty understanding or respecting rights of others	51	41	63	0.27	56	0.26
9[f]	Difficulty in group activities, invents own rules	64	71	75	0.43	55	0.36
0	Difficulty finding friends	42	59	55	0.12	39	0.12
1	Has little interaction with age peers	37	46	60	0.18	49	0.20
2	Difficulty in group games	54	73	72	0.42	52	0.32
3	Is not accepted into children's games	53	56	68	0.35	47	0.26
4	Is not interested in physical contact	21	35	72	0.32	68	0.35
5[f]	Has one/few interests that impinge	37	44	65	0.27	51	0.25
6[f]	Repeats/gets stuck in meaningless things	30	39	70	0.34	61	0.30
7[f]	Is very upset by tiny routine change	45	69	69	0.40	60	0.44
8[f]	Different eye gaze	9	37	65	0.10	61	0.08

em #	Item definition	Distribution		Agreement			
		Any difficulties (score 1 or 2)		Dichotomous use of scale (0/1–2)		Full use of scale (0/1/2)	
		Original scoring %	Retrospective Scoring %	Equal score both times %	Kappa	Equal score both times %	Weighted kappa
	Internalized emotional/behavioural problems						
9[g]	Has poor self-confidence	59	63	70	0.37	46	0.24
0[g]	Is unhappy, sad, depressed	32	51	66	0.33	58	0.28
1[g]	Has feelings of loneliness	20	33	63	0.06	59	0.09
2[g]	Has tried to inflict bodily harm to self	14	23	81	0.39	80	0.37
3[g]	Has poor appetite	21	16	83	0.42	78	0.32
4[g]	Feels worthless	32	38	65	0.24	57	0.15
5[f]	Complains of bellyaches, headaches etc.	31	36	71	0.35	64	0.28
6[f]	Appears tense and worried	27	34	63	0.13	61	0.10
7[f]	Worries on leaving home	12	36	66	0.13	64	0.16
8[f]	Sleeps less than other children	16	36	79	0.49	71	0.38
9[f]	Has nightmares	12	26	72	0.13	70	0.19
0[f]	Has sleepwalking or other night-time attacks	8	10	85	0.10	85	0.08
	Externalized emotional/behavioural problems						
1[g]	Loses temper	69	54	75	0.49	42	0.27
2[g]	Argues with adults	43	37	69	0.36	57	0.25
3[g]	Refuses to follow adult`s instructions	57	46	65	0.30	51	0.23
4[g]	Teases others	44	34	81	0.61	66	0.44
5[g]	Often blames others for own mistakes	69	41	59	0.23	41	0.23
6[g]	Often slighted or disturbed by others	56	59	72	0.42	55	0.34
7[g]	Often in a fight	29	26	78	0.46	69	0.35

em #	Item definition	Distribution		Agreement			
		Any difficulties (score 1 or 2)		Dichotomous use of scale (0/1–2)		Full use of scale (0/1/2)	
		Original scoring %	Retrospective Scoring %	Equal score both times %	Kappa	Equal score both times %	Weighted kappa
8[g]	Is cruel to animals	8	5	91	0.20	91	0.18
9[g]	Lies and cheats	36	24	76	0.44	71	0.43
0[g]	Steals things at home	8	9	92	0.50	92	0.39
1[g]	Often destroys family members' things	20	14	82	0.35	78	0.25
2[f]	Has episodes of extremely high activity	16	21	74	0.13	72	0.16
3[f]	Has recurrent episodes of obvious irritability	35	33	74	0.42	70	0.42
	Obsessive-compulsive emotional/behavioural problems						
4[f]	Compulsively repeating certain activities	32	39	75	0.46	67	0.50
5[g]	Has obsessive/fixed ideas	17	30	70	0.18	63	0.14
6[g]	Has uncontrollable movements, tics etc.	17	31	65	0.05	62	0.08
7[g]	Repeats meaningless movements	16	28	72	0.20	67	0.17
8[g]	Emits unmotivated sounds	22	25	81	0.48	75	0.42
9[g]	Difficulty keeping quiet	38	27	70	0.32	63	0.24
0[g]	Repeats words/parts of words	6	5	95	0.55	95	0.58
1[g]	Uses dirty words in exaggerated fashion	31	16	82	0.52	73	0.42

a. *n*=48.

b. *n*=58.

c. *n*=55.

d. *n*=65.

e. *n*=67.

f. *n*=60.

g. n=66.
