# Supplementary material for: Retrospective parental assessment of childhood neurodevelopmental problems: the use of the Five to Fifteen questionnaire in adults
Source: BJPsych Open. 2019 May 17;5(3):e42. doi: 10.1192/bjo.2019.30 (PMC6537455; doi:10.1192/bjo.2019.30)
Supplement: Supplementary file 1 [file bjosup.zip › S2056472419000309sup001.zip!/S2056472419000309sup001/Table S1 1 English FTF Brief.pdf]

	*Five to fifteen – Brief	Does not apply	Applies sometimes or to some extent	Definitely applies
1	Difficulty acquiring new motor skills such as biking, skating, swimming			
2	Difficulty running fast and smoothly			
3	Difficulty using cutlery			
4	Often fails to give attention to details, or makes careless mistakes			
5	Is easily distracted (e.g., by irrelevant sounds such as others talking, cars driving past etc)			
6	Is constantly moving in some way (fidgets with hands or feet)			
7	Is constantly "on the go", or things are done too hurriedly			
8	Difficulty completing tasks, doesn't finish things as others do			
9	Difficulty planning completion of tasks (e.g., to bring along things needed for an outing or for school)			
10	Does not care about the fit of clothes, does not adjust socks or pants that slide down, etc.			
11	Difficulty managing jig-saw puzzles			
12	Difficulty learning things by heart, e.g., songs, nursery rhymes, multiplication tables, etc.			
13	Difficulty understanding explanations and instructions			
14	Difficulty pronouncing complex words such as rabbit, binoculars, or spaghetti			
15	Difficulty explaining what has happened (e.g., describing what he / she did during the day, or on vacation, so that the listener understands his / her experience)			
16	Difficulty understanding what he/she is reading			
17	Difficulty with maths problems formulated as written text			
18	Trouble persisting and completing tasks, often leaving them unfinished			
19	Perceived as odd, especially by age peers			
20	Says socially inappropriate things			
21	Gets upset by minor changes to daily routines			
22	Often loses temper			
23	Often annoys others by being deliberately provocative			
24	Repeats certain actions compulsively, or has habits that are very difficult to change			

*FTF-Brief consists of 24 items representing 18 of the original 22 subdomains

- Kadesjö B, Janols L-O, Korkman M, Mickelsson K, Strand G, Trillingsgaard A, et al. The FTF (Five to Fifteen): the development of a parent questionnaire for the assessment of ADHD and comorbid conditions. *Eur Child Adolesc Psychiatry*. 2004;13 Suppl 3:3-13.
- Lugnegård T, Bejerot S. Retrospective parental assessment of childhood neurodevelopmental problems: the use of the "Five To Fifteen" in adults. BJPOpen, 2019.
