# Supplementary material for: Retrospective parental assessment of childhood neurodevelopmental problems: the use of the Five to Fifteen questionnaire in adults
Source: BJPsych Open. 2019 May 17;5(3):e42. doi: 10.1192/bjo.2019.30 (PMC6537455; doi:10.1192/bjo.2019.30)
Supplement: Supplementary file 1 [file bjosup.zip › S2056472419000309sup001.zip!/S2056472419000309sup001/Table S2 Swedish FTF Brief .pdf]

	*Five to Fifteen - Brief	Stämmer inte	Stämmer ibland/ I viss mån	Stämmer bra
1	Har svårt för att lära sig nya motoriska färdigheter som att cykla, åka skridskor, simma			
2	Har svårt att springa snabbt och smidigt			
3	Har svårt för att använda kniv och gaffel			
4	Är ofta ouppmärksam på detaljer eller gör slarvfel			
5	Blir lätt distraherad eller störd (tex av ovidkommande ljud som att andra pratar, bilar kör förbi etc)			
6	Är ständigt i rörelse på något sätt (vänder och vrider sig, trummar med fingrarna, pillar med något etc)			
7	Är ständigt i ”full fart” eller det blir ofta alltför högt tempo i det han/hon gör			
8	Har svårt att avsluta en uppgift/aktivitet, blir inte färdig som de andra			
9	Har svårt att planera utförandet av en aktivitet (tex att få med sig alla saker som behövs för en utflykt eller det som skall med till skolan)			
10	Bryr sig inte om hur kläderna sitter på kroppen, rättar inte till strumpor eller byxor som glider ner etc.			
11	Har svårt att lägga pussel			
12	Har svårt att lära sig ramsor, sånger, multiplikationstabellen utantill etc.			
13	Har svårt att förstå förklaringar och instruktioner			
14	Har svårt att uttala krångliga ord, tex ”elektriskt”, ”rutschkana”			
15	Har svårt att berätta om upplevelser eller förklara samman-hang (tex att berätta vad han/hon gjort under dagen eller på sommarlovet så att lyssnaren förstår vad han/hon varit med om)			
16	Har svårt att förstå vad han/hon läser			
17	Har svårt klara benämnda tal dvs räkneuppgifter där han/hon måste läsa en text			
18	Har svårt att hålla fast vid och göra färdigt en uppgift, lämnar den ofta halvfärdig			
19	Uppfattas som annorlunda, udda, speciell av jämnåriga			
20	Säger socialt opassande saker			
21	Blir mycket oroad av små förändringar i de dagliga rutinerna			
22	Förlorar ofta humöret			
23	Retar ofta andra genom att medvetet göra saker som upplevs provocerande			
24	Upprepar på ett tvångsmässigt sätt handlingar eller har vanor som är mycket svåra att ändra på			

*FTF-Brief är en förkortad version av Fem till Femton och inkluderar 24 item som representerar 18 av de 22 ursprungliga subdomänerna i FTF.

- Kadesjö B, Janols L-O, Korkman M, Mickelsson K, Strand G, Trillingsgaard A, et al. The FTF (Five to Fifteen): the development of a parent questionnaire for the assessment of ADHD and comorbid conditions. *Eur Child Adolesc Psychiatry*. 2004;13 suppl 3:3-13;
- Lugnegård T, Bejerot S. Retrospective parental assessment of childhood neurodevelopmental problems: the use of the “Five To Fifteen” in adults. *BJPsych Open,* 2019.
